# Disparities in race/ethnicity and socioeconomic status: risk of mortality of breast cancer patients in the California Cancer Registry, 2000–2010

**DOI:** 10.1186/1471-2407-13-449

**Published:** 2013-10-02

**Authors:** Carol A Parise, Vincent Caggiano

**Affiliations:** 1 Sutter Institute for Medical Research, 2801 Capitol Ave Suite 400, Sacramento, CA 95816, USA

**Keywords:** Disparities, Breast cancer-specific mortality, Race/ethnicity, Socioeconomic status

## Abstract

**Background:**

Racial disparities in breast cancer survival have been well documented. This study examines the association of race/ethnicity and socioeconomic status (SES) on breast cancer-specific mortality in a large population of women with invasive breast cancer.

**Methods:**

We identified 179,143 cases of stages 1–3 first primary female invasive breast cancer from the California Cancer Registry from January, 2000 through December, 2010. Cox regression, adjusted for age, year of diagnosis, grade, and ER/PR/HER2 subtype, was used to assess the association of race/ethnicity on breast cancer-specific mortality within strata of stage and SES. Hazard ratios (HR) and 95% confidence intervals were reported.

**Results:**

Stage 1: There was no increased risk of mortality for any race/ethnicity when compared with whites within all SES strata. Stage 2: Hispanics (HR = 0.85; 0.75, 0.97) in the lowest SES category had a reduced risk of mortality.. Blacks had the same risk of mortality as whites in the lowest SES category but an increased risk of mortality in the intermediate (HR = 1.66; 1.34, 2.06) and highest (HR = 1.41; 1.15, 1.73) SES categories. Stage 3: Hispanics (HR = 0.74; 0.64, 0.85) and APIs (HR = 0.64; 0.50, 0.82) in the lowest SES category had a reduced risk while blacks had similar mortality as whites. Blacks had an increased risk of mortality in the intermediate (HR = 1.52; 1.20, 1.92) and highest (HR = 1.53; 1.22, 1.92) SES categories.

**Conclusions:**

When analysis of breast cancer-specific mortality is adjusted for age and year of diagnosis, ER/PR/HER2 subtype, and tumor grade and cases compared within stage and SES strata, much of the black/white disparity disappears. SES plays a prominent role in breast cancer-specific mortality but it does not fully explain the racial/ethnic disparities and continued research in genetic, societal, and lifestyle factors is warranted.

## Background

Breast cancer is the most common cancer in women residing in California, regardless of age or race/ethnicity [1-3] but the burden of this cancer has an unequal racial/ethnic distribution. Racial disparities in breast cancer incidence and mortality have been well documented in the past, particularly among African American women, who have been found to have a lower incidence of breast cancer compared to white women, but a higher overall mortality [4,5].

A wealth of studies have documented the many factors specifically associated with disparities of cancer care such as age, race/ethnicity, socioeconomic status (SES), access to health care, cultural, medical, and health provider issues [6-18]. Additionally, prognostic factors directly related to breast cancer including tumor size, histology, grade, nodal and receptor status, and stage at diagnosis are expressed differentially in the population by age and race/ethnicity [19-23] adding further complexity to any discussion of disparities in cancer care.

Over 40 years ago, the California Cancer Registry (CCR) noted that breast cancer patients treated at private hospitals survived their cancer better than patients treated in public hospitals [24]. Expanding on this early attempt to explain how social class or SES relates to breast cancer survival, the objective of this present investigation is to determine if the association of race/ethnicity on breast cancer survival persists when analyses are conducted that compare patients within the same SES category and stage at diagnosis.

## Methods

Using the population-based CCR, we identified cases of American Joint Commission on Cancer (AJCC) stages 1–3 first primary female invasive breast cancer (ICDO-3 sites C50.0-C50.9) [25] diagnosed between January 1, 2000 through December 31, 2010 and reported to the CCR as of January, 2012. Cases are reported to the Cancer Surveillance Section of the California Department of Public Health from hospitals and any other facilities providing care or therapy to cancer patients residing in California [26]. Cases identified outside of California, only at autopsy, or from death certificates were excluded. Breast cancer-specific mortality was defined as a death due to breast cancer as documented by the codes ranging from C50.01 to C50.91 of the International Statistical Classification of Diseases and Related Health Problems, 10th Revision. Deaths due to causes other than cancer were censored.

### SES

Quintile of SES was derived using data from the 2000 U.S. census. SES was assigned at the census block group level and based on address at time of initial diagnosis, as reported in the medical record. This area based composite SES measure was created through principal components analysis [27] and included the following census variables: proportion with a blue-collar job, proportion older than 16 years without a job, median household income, population living below 200% Federal Poverty Level, median gross rent, median value of owner-occupied houses, and a median education index [28]. Quintiles of SES ranging from 1 (the lowest/ least affluent) to 5 (the highest/most affluent) were computed. This area based SES measure has been used in many studies utilizing cancer registry data [22,29-33]. A detailed description of this methodology is found in other publications [34].

For ease of presentation, in this study, we combined the lowest two quintiles 1 + 2 (lowest/least affluent) as well as the highest two quintiles 4 + 5 (highest/most affluent).The intermediate (3) remained intact.

### Race/ethnicity

Race/ethnicity was classified into six distinct categories: White, African American or black, Hispanic, Asian-Pacific Islander (API), American Indian, and Hispanic plus other race. The race/ethnicity information contained in the medical record was obtained by patient self-identification, assumptions based on personal appearance, or inferences based on the race/ethnicity of the parents, birthplace, surname, or maiden name. The API category was derived from combining cases identified as Pacific Islander, Southeast Asian, Indian continent, Chinese, Japanese, Filipino, and Korean.

Determination of Hispanic ethnicity was based on information from the medical record and computer-based comparisons to the 1980 U.S. census list of Hispanic surnames. Patients identified as Hispanic on the medical record as white with a Hispanic surname were classified as Hispanic. Cases identified as black or API and also identified as Hispanic were categorized as Hispanic plus other race. This classification resulted in six mutually exclusive categories: White, black, Hispanic, API, American Indian, and Hispanic plus other race.

### ER/PR/HER2

The details of documentation of estrogen receptor (ER) progesterone receptor (PR), and human epidermal growth factor receptor 2 (HER2) along with age and stage at diagnosis, and tumor grade have been extensively described in our previous publications [22,32,35,36]. And by the CCR [26]. Age was grouped into five categories (<35, 35–69, 70–79, 80–89, and 90+ years). Year of diagnosis was categorized as 2000–2006 and 2007–2010. The year 2007 marks the first complete year following approval of trastuzumab for adjuvant therapy for breast cancer.

### Statistical analysis

The number of cases with missing data for ER, PR, HER2, race, grade, cause of death, and survival time were computed. Contingency tables were used to assess the distribution of missing data by race, and the distribution of demographic and tumor characteristics among SES strata.

Cox proportional hazards modeling was used to determine time from breast cancer diagnosis to time of breast cancer-specific death for African Americans, Hispanics, and APIs, when compared with whites. All analyses were conducted separately for each stage because of the differences in prognosis of patients diagnosed in different stages. Separate models with and without SES were run to test whether SES confounded the association of race with mortality. The interaction between race and SES was then tested to determine if the affect of race on mortality varied among the levels of SES.

Analyses were stratified by both stage and SES so that the risk of mortality for each race could be estimated for cases within each stage/SES stratum.

All models were adjusted for age, ER/PR/HER2, grade, and year of diagnosis. Hazard ratios (HR) and 95% Confidence Intervals (CIs) were computed for all models. The HR represents the estimated risk of mortality for two people of the same age and tumor characteristic when one person is black, Hispanic, or API, and the other person is white.

This research study involved analysis of existing data from the California Cancer Registry without subject intervention. No identifiers were linked to subjects. Therefore, the study was approved by Sutter Health Central Institutional Review Committee under the category "exempt".

## Results

### Missing data

The initial data contained 181,090 cases of stages 1–3 first primary invasive breast cancer. Cases where race was identified as American Indian (n = 275), Hispanic plus other race (n = 517), or race unknown (n = 1,155) were excluded, which resulted in 179,143 cases with complete data for year of diagnosis, age, race/ethnicity, stage, vital status, and SES. Tumor grade, ER/PR/HER2 status, and unknown cause of death were missing for 57,695 cases leaving 123,395 cases with complete data (Table 1).

**Table 1:** Summary of missing data for incident female stages 1–3 invasive breast cancer reported to the California Cancer Registry 2000–2010 with complete data for age, SES, year of diagnosis, survival time, and race/ethnicity (N = 179,143)

**Missing**	**n(%)**
ER	16,247 (9.0%)
PR	21,306 (11.8%)
HER2*	45,021 (24.9%)
Tumor grade	10,594 (5.9%)
Unknown cause of death	2,386 (1.5%)
**Total cases with one or more of the above variables missing**^**†**^	**57,695 (31.9%)**
**Total cases with complete data**	**123,395 (68.1%)**

^*^HER2 data not easily retrievable in the registry until 2006.

^†^Cases may be missing data for more than one variable.

The distribution of missing ER, PR and HER2 was similar for all race/ethnicities ranging from 8.2% to 9.8% for ER; 10.9% to 12.6% for PR and 24.4% to 25.9% for HER2. There was little variation among the race/ethnicities for missing grade, ranging from 5.5% to 6.0%. Cause of death was missing equally among all races (1.2% to 1.5%).

### Demographics and tumor characteristics

Table 2 shows the distribution of cases within each of the SES categories. There was no appreciable difference in the year of diagnosis among all SES categories. Of the patients within the lowest SES category, 50.2% were white, 10.9% were black, 29.8% were Hispanic, and 9.1% were API. In contrast, within the highest SES category, the percents were 76.1%, 3.1%, 8.4%, and 12.4% respectively. Over 50% of African Americans and Hispanics were in the lowest and intermediate SES categories.

**Table 2:** Distribution of demographic and tumor characteristics of stages 1–3 first primary invasive breast cancer by socioeconomic status: California Cancer Registry 2000–2010*

		**Socioeconomic status category**			
		**Lowest/least affluent**	**Intermediate**	**Highest/most affluent**	**Total**
**N (%)**		49,868 (27.8%)	37,128 (20.7%)	92,147 (51.36%)	**179,143**
**Year at diagnosis**					
2000-2006	n	31,380	23,844	59,359	114,583
	% within SES	62.9%	64.2%	64.4%	64.0%
2007-2010	n	18,488	13,284	32,788	64,560
	% within SES	37.1%	35.8%	35.6%	36.0%
**Race/ethnicity**					
White	n	25,016	25,283	70,136	120,435
	% within SES	50.2%	68.1%	76.1%	67.2%
Black	n	5,454	2,349	2,834	10,637
	% within SES	10.9%	6.3%	3.1%	5.9%
Hispanic	n	14,848	5,756	7,776	28,380
	% within SES	29.8%	15.5%	8.4%	15.8%
API	n	4,550	3,740	11,401	19,691
	% within SES	9.1%	10.1%	12.4%	11.0%
**Age (years) at diagnosis**					
<35	n	1,347	713	1,628	3,688
	% within SES	2.7%	1.9%	1.8%	2.1%
35-69	n	35,557	25,924	66,589	128,070
	% within SES	71.3%	69.8%	72.3%	71.5%
70-79	n	8,414	6,708	15,388	30,510
	% within SES	16.9%	18.1%	16.7%	17.0%
80-89	n	4,022	3,349	7,565	14,936
	% within SES	8.1%	9.0%	8.2%	8.3%
90+	n	528	434	977	1,939
	% within SES	1.1%	1.2%	1.1%	1.1%
**AJCC stage**					
Stage 1	n	21,814	18,066	47,655	87,535
	% within SES	43.7%	48.7%	51.7%	48.9%
Stage 2	n	21,082	14,776	35,550	71,408
	% within SES	42.3%	39.8%	38.6%	39.9%
Stage 3	n	6,972	4,286	8,942	20,200
	% within SES	14.0%	11.5%	9.7%	11.3%
**ER/PR/HER2 subtype**					
ER+/PR+/HER2-	n	18,434	15,088	40,900	74,422
	% within SES	52.6%	56.6%	59.8%	57.2%
ER+/PR+/HER2+	n	3,521	2,519	6,204	12,244
	% within SES	10.0%	9.5%	9.1%	9.4%
ER+/PR-/HER2-	n	3,264	2,548	6,702	12,514
	% within SES	9.3%	9.6%	9.8%	9.6%
ER+/PR-/HER2+	n	1,180	856	2,082	4,118
	% within SES	3.4%	3.2%	3.0%	3.2%
ER-/PR+/HER2-	n	285	212	527	1,024
	% within SES	0.8%	0.8%	0.8%	0.8%
ER-/PR+/HER2+	n	167	120	226	513
	% within SES	0.5%	0.5%	0.3%	0.4%
ER-/PR-/HER2-	n	5,454	3,550	7,821	16,825
	% within SES	15.6%	13.3%	11.4%	100.0%
ER-/PR-/HER2+	n	2,744	1,753	3,941	12.9%
	% within SES	7.8%	6.6%	5.8%	8,438
**Tumor grade**	n	9,276	7,998	22,058	39,332
Grade I	% within SES	19.9%	22.9%	25.3%	23.3%
	n	18,814	14,625	37,837	71,276
Grade II	% within SES	40.3%	41.9%	43.4%	42.3%
	n	17,586	11,572	25,888	55,046
Grade III	% within SES	37.7%	33.2%	29.7%	32.6%
	n	1,001	698	1,344	3,043
Grade IV	% within SES	2.1%	2.0%	1.5%	1.8%

*Excludes cases classified as American Indian and Hispanic + Other race.

The relationship between stage and SES was informative. For patients in the lowest SES category, 43.7% were stage 1, whereas 51.7% of patients within the highest SES category had stage 1 disease. For stages 2 and 3, with each increase in an SES category a decrease in the percent of patients was noted.

A significantly higher percent of patients within the lowest SES category had the ER-/PR-HER2- and ER-/PR-HER2+ subtypes when compared with the highest SES category (Table 2).

The distribution of cases by race/ethnicity is shown in Table 3. The majority of white (58.2%) and API patients (57.9%) were in the highest SES category. In contrast, the majority of black (51.3%) and Hispanic patients (52.3%) were in the lowest SES category. Of the 3,688 patients under 35 years of age, 1,600 (43.4%) were white and 1,215 (32.9%) were Hispanic. However, only 1.3% of white patients were less than 35 years of age, whereas 4.3% of Hispanic patients were in that age group. With each increasing age category, the percent of whites diagnosed increased progressively while the percent of all other races decreased. Over 80% of women aged 80 and older were white.

**Table 3:** Distribution of demographic and tumor characteristics of stages 1–3 first primary invasive breast cancer by race/ethnicity: California Cancer Registry 2000–2010*

		**Race/Ethnicity**				
		**White**	**Black**	**Hispanic**	**API**	**Total**
**N (%)**		120,435 (67.2%)	10,637 (5.9%)	28,380 (15.8%)	19,691 (11.1%)	**179,143**
**Year at diagnosis**						
	n	79,226	6,662	17,054	11,641	114,583
2000-2006	% within race/ethnicity	65.8%	62.6%	60.1%	59.1%	64.0%
	n	41,209	3,975	11,326	8,050	64,560
2007-2010	% within race/ethnicity	34.2%	37.4%	39.9%	40.9%	36.0%
**Socioeconomic status**						
	n	25,016	5,454	14,848	4,550	49,868
Lowest/Least Affluent	% within race/ethnicity	20.8%	51.3%	52.3%	23.1%	27.8%
	n	25,283	2,349	5,756	3,740	37,128
Intermediate	% within race/ethnicity	21.0%	22.1%	20.3%	19.0%	20.7%
	n	70,136	2,834	7,776	11,401	92,147
Highest/Most Affluent	% within race/ethnicity	58.2%	26.6%	27.4%	57.9%	51.4%
**Age at diagnosis (years)**						
	n	1,600	299	1,215	574	3,688
<35	% within race/ethnicity	1.3%	2.8%	4.3%	2.9%	2.1%
	n	81,900	8,055	22,331	15,784	128,070
35-69	% within race/ethnicity	68.0%	75.7%	78.7%	80.2%	71.5%
	n	23,132	1,518	3,466	2,394	30,510
70-79	% within race/ethnicity	19.2%	14.3%	12.2%	12.2%	17.0%
	n	12,223	672	1,190	851	14,936
80-89	% within race/ethnicity	10.1%	6.3%	4.2%	4.3%	8.3%
	n	1,580	93	178	88	1,939
90+	% within race/ethnicity	1.3%	0.9%	0.6%	0.4%	1.1%
**AJCC stage**	n	62,693	4,195	11,321	9,326	87,535
Stage 1	% within race/ethnicity	52.1%	39.4%	39.9%	47.4%	48.9%
	n	45,791	4,751	12,608	8,258	71,408
Stage 2	% within race/ethnicity	38.0%	44.7%	44.4%	41.9%	39.9%
	n	11,951	1,691	4,451	2,107	20,200
Stage 3	% within race/ethnicity	9.9%	15.9%	15.7%	10.7%	11.3%
**ER/PR/HER2 subtype**						
	n	52,797	3,240	10,526	7,859	74,422
ER+/PR+/HER2-	% within race/ethnicity	60.3%	42.9%	51.0%	54.9%	57.2%
	n	7,689	709	2,181	1,665	12,244
ER+/PR+/HER2+	% within race/ethnicity	8.8%	9.4%	10.6%	11.6%	9.4%
	n	8,791	735	1,816	1,172	12,514
ER+/PR-/HER2-	% within race/ethnicity	10.0%	9.7%	8.8%	8.2%	9.6%
	n	2,675	252	678	513	4,118
ER+/PR-/HER2+	% within race/ethnicity	3.1%	3.3%	3.3%	3.6%	3.2%
	n	640	77	194	113	1,024
ER-/PR+/HER2-	% within race/ethnicity	0.7%	1.0%	0.9%	0.8%	0.8%
	n	281	45	126	61	513
ER-/PR+/HER2+	% within race/ethnicity	0.3%	0.6%	0.6%	0.4%	0.4%
	n	9,924	1,929	3,371	1,601	16,825
ER-/PR-/HER2-	% within race/ethnicity	11.3%	25.5%	16.3%	11.2%	12.9%
	n	4,789	572	1,748	1,329	8,438
ER-/PR-/HER2+	% within race/ethnicity	5.5%	7.6%	8.5%	9.3%	6.5%
**Tumor grade**	n	29,749	1,516	4,613	3,454	39,332
Grade I	% within race/ethnicity	26.2%	15.1%	17.3%	18.6%	23.3%
	n	49,124	3,570	10,610	7,972	71,276
Grade II	% within race/ethnicity	43.3%	35.5%	39.8%	43.0%	42.3%
	n	32,747	4,691	10,823	6,785	55,046
Grade III	% within race/ethnicity	28.9%	46.7%	40.6%	36.6%	32.6%
	n	1,802	276	620	345	3,043
Grade IV	% within race/ethnicity	1.6%	2.7%	2.3%	1.9%	1.8%

*Excludes cases classified as American Indian and Hispanic + Other race.

The ER+/PR+/HER2- subtype was the most common (57.2%), but variation by race/ethnicity was noted, especially between white (60.3%) and black patients (42.9%). Black and Hispanic patients had the highest percent of the triple-negative subtype, 25.5% and 16.3%, respectively. Whites had the lowest percent of patients among the four HER2-positive subtypes. This was especially noticeable within the ER-/PR-/HER2+ subtype, the molecularly defined HER2-overexpressing subtype, with whites having the lowest (5.5%) and API patients the highest (9.3%) percent.

The majority of white patients (52.1%) presented in stage 1, compared with approximately 40% of both black and Hispanic patients presenting in this stage. A higher percent of black (15.9%) and Hispanic (15.7%) patients presented with stage 3 disease compared with white (9.9%) and API (10.7%) patients. Over 60% of white patients presented with the ER+/PR + HER2- subtype. Among black patients, 25.5% had ER-/PR-HER2- compared with only 11.3% of whites. African Americans, Hispanics, and API patients were diagnosed at a higher grade (Table 3).

### Cox proportional hazards

Cox proportional hazards models adjusted for age, ER/PR/HER2, grade, and year of diagnosis indicated that inclusion of SES was a confounder of the association of race with breast cancer-specific mortality (results not shown). SES reduced the effect of all race/ethnicities on mortality in all stages. The strength of the effect of SES was strongest for blacks in stage 1 where the HR was reduced 9.2% from 1.32 for blacks without inclusion of SES to 1.19 when included. The models that included the interaction between SES and race/ethnicity were statistically significant for stages 2 and 3 (p < 0.05) which indicated that the association of race with mortality was not the same for all levels of SES. Therefore models stratified by both stage and SES were more appropriate and these results are presented in Table 4.

**Table 4:** Hazard ratios and 95% confidence intervals derived from Cox regression for race/ethnicity after adjustment for age, year of diagnosis, grade, and ER/PR/HER2 subtype*

**Stage 1**	**HR (95% CI)**
**SES**	
Lowest/least affluent (n = 14,011)	
White	1.00
Black	1.19 (0.85, 1.65)
Hispanic	0.96 (0.74, 1.13)
API	0.69 (0.43, 1.11)
Intermediate (n = 11,839)	
White	1.00
Black	0.88 (0.51, 1.54)
Hispanic	0.93 (0.64, 1.36)
API	0.92 (0.59, 1.43)
Highest/most affluent (n = 32,945)	
White	1.00
Black	1.47 (0.96, 2.27)
Hispanic	1.05 (0.76, 1.44)
API	0.84 (0.63, 1.13)
Stage 2	
SES	
Lowest/least affluent (14,063)	
White	1.00
Black	1.14 (0.97, 1.38)
Hispanic	0.85 (0.75, 0.97)
API	0.85 (0.69, 1.04)
Intermediate (10,141)	
White	1.00
Black	1.66 (1.34, 2.06)
Hispanic	1.11 (0.92, 1.32)
API	0.80 (0.62, 1.03)
Highest/most affluent (25,323)	
White	1.00
Black	1.41 (1.15, 1.73)
Hispanic	1.12 (0.95, 1.31)
API	0.92 (0.79, 1.07)
Stage 3	
SES	
Lowest/least affluent (4,805)	
White	1.00
Black	1.05 (0.88, 1.25)
Hispanic	0.74 (0.64, 0.85)
API	0.64 (0.50, 0.83)
Intermediate (3,087)	
White	1.00
Black	1.52 (1.20, 1.92)
Hispanic	1.12 (0.91, 1.37)
API	0.92 (0.69, 1.23)
Highest/most affluent (6,532)	
White	1.00
Black	1.53 (1.22, 1.92)
Hispanic	0.97 (0.79, 1.17)
API	0.92 (0.76, 1.11)

*Confidence intervals that include 1.00 indicate that the risk of mortality for a race/ethnicity was not statistically significantly better or worse than for whites within a stage/SES stratum.

Table 4 shows that in stage 1 there was no increased risk of mortality for any race/ethnicity when compared with whites for all SES categories. In stage 2, Hispanics had a 15% reduced risk of mortality in the lowest SES category. Blacks had the same risk of mortality as whites in lowest SES category. However, in the intermediate and highest SES categories, blacks had a statistically significantly higher risk of mortality.

For stage 3, in the lowest SES category, Hispanics and APIs had a reduced risk of mortality while blacks had similar mortality as whites. In the intermediate SES category, blacks had a 52% increased risk of mortality and a 53% increased risk in the highest SES category.

For all stages, there was no black/white disparity in the lowest SES category. However, Hispanics in the lowest SES had better survival than whites in stages 2 and 3.

## Discussion

Racial disparities in breast cancer treatment and outcomes have been previously well documented [2,8,37,38]. Survival differences between African American and white patients with breast cancer have often been attributed to more advanced stage at diagnosis [39], unfavorable tumor biology features such as hormone receptor-negative disease [19] or triple-negative disease [40], lower SES [5,41], and inferior use of adjuvant treatments [9,42-46].

It remains difficult to completely separate and untangle the interplay among race/ethnicity, SES, and tumor biology, and determine their respective roles in breast cancer outcomes. This dilemma is evident from the conflicting results of studies investigating racial/ethnic disparities in cancer. Some have shown comparable outcomes after adjustment for sociodemographic factors if patients have equal access to healthcare [47-52]. Others have found that low SES, not race, was associated with poorer outcomes [41,53,54].

Further, some studies have shown racial disparities even after adjusting for SES. In a meta-analysis of 20 studies representing a total of 14,013 African Americans and 76,111 white American women diagnosed with breast cancer from 1961 to 2003, Newman concluded that African American ethnicity is a significant and independent predictor of poor outcome from breast cancer, even after accounting for SES [55]. Also, a Southwest Oncology Group study concluded that, after adjustment for SES, African American patients with breast cancer had worse adjusted survival, despite enrollment on phase III clinical trials with uniform stage, treatment, and follow-up [56]. These latter studies, as well as others [57-59] suggest biologic differences in tumor behavior as the reason for racial/ethnic disparities.

Others argue against a biologic hypothesis for racial disparities. In a study of breast cancer-specific mortality rates for women in Chicago, New York City, and the United States from 1980–2005, race-specific rate ratios were used to measure the disparity in breast cancer-specific mortality. In all three locations the black and white rates were similar in the 1980s and remained that way until the 1990s, when the white rates started to decline while the black rates remained constant, just as the benefits from early detection by mammography and from treatment were noticeable [60-62]. These findings seem to argue against differential tumor biology.

The goal of our present study was to assess racial/ethnic disparities within three levels of SES and within the same stage of disease so that variability among treatment and access to care would be minimized. We also adjusted for ER/PR/HER2 because of the known propensity of African American and Hispanic women to have hormone receptor-negative and, in particular, triple-negative phenotype [16,19,22,40].

The present investigation has shown that for women with stage 1 breast cancer, there is no disparity among any race/ethnicity regardless of the SES category. In addition, there is no black/white disparity within the lowest SES category regardless of stage of disease, but a disparity is apparent in the higher SES categories. African Americans in the intermediate and highest SES categories with stages 2 and 3 breast cancer have increased risk of mortality when compared with whites. Interestingly, low SES Hispanic patients with stages 2 and 3 disease have a lower risk of mortality when compared to low SES white patients, similar to what has been described in the "Hispanic Paradox" [63].

As is often the case, a correlational study raises more questions than answers. On the one hand, a differential tumor or host biology does not seem to be plausible because there were no differences in risk of mortality among any race/ethnicity in stage 1 and there was no black/white disparity for women in the lowest SES category regardless of stage. On the other hand, for higher stages of disease, black patients in the same, higher SES category had an increased risk of mortality as compared to white patients while Hispanics in the lowest SES category at higher stages had decreased risk of mortality as did APIs in Stage 3.

The findings of this study raise the question of whether tumor or host factors play a role in advanced stages of disease. Do black, white, Hispanic, and API patients respond differentially to treatments? Data regarding racial/ethnic differences in the pharmcogenomics of chemotherapy and endocrine response and toxicities are limited [64-66]. Alternatively, are more aggressive treatments offered or available to patients of all race/ethnicities even when there is presumed equal access to care? Is there an element of racial/ethnic discrimination in receipt of more aggressive cancer treatments [62,67,68]?

The results of this population-based registry study cannot definitively answer these perplexing questions, but at least in stage 1 disease, a differential tumor biology appears unlikely. It also appears that SES plays a prominent role in cancer outcomes although genetic, environmental, societal, lifestyle, and health provider factors may also contribute to racial disparities, and they should not be overlooked [69].

The limitations of population-based cancer registry investigations including exclusion of subjects without ER, PR, and HER2 are well known [22,32,45,70-72]. Accurate and precise treatment information was not available from the registry. Although it has been suggested that suboptimal use of adjuvant treatments may explain differences in outcomes, [9,42-46] others have reported little or no differences between black and white patients with regard to chemotherapy administration [73-75]. Differences in adjuvant treatment between black and white women may explain the disparities we noted in stages 2 and 3 [76,77]. However, since the disparities occurred only in the two highest SES categories, we can speculate that patients of all race/ethnicities should have had equal access to adjuvant treatment.

We recognize that determination of race/ethnicity can be problematic and arbitrary. Hispanic ethnicity may include women from Mexico, Central and South America, Spain, as well as Puerto Rico and Cuba. The category API may include women from Asia, the Indian Continent, and the Pacific Islands. We also recognize that our measure of SES was at the neighborhood level rather than at the individual level. The CCR does not obtain the information necessary to determine individual SES but others have commented on the usefulness of composite SES measures [78,79] In addition, this measure of SES has been used in many studies that utilize cancer registry data [22,29-33].

Lastly, other than age, we have no information about reproductive history and lifestyle risk factors such as nulliparity, multiparity, breast feeding, diet, body fat distribution, use of alcohol, oral contraceptives, or hormone replacement treatments that may determine the type of breast cancer and ultimately impact survival, [80-88].

Despite these shortcomings, our study is unique because of the large number of cases reported to the statewide cancer registry from an ethnically diverse population. Unlike other studies that employed different methodologies of SES [55] or had extensive missing SES information [56,89,90], we used a validated measure of SES for all 179,143 patients and most importantly, we stratified by both stage and SES to minimize the potential that our results would be due to differences either in severity of disease or access to care.

## Conclusions

Our research has shown that when breast cancer-specific mortality is analyzed either by race/ethnicity or by SES, significant differences exist among the races with respect to age at presentation, stage at diagnosis, ER/PR/HER2 subtype, and tumor grade. However, when adjusting analyses for these variables and comparing cases within stage and SES strata, much of the black/white disparity disappears.

SES plays a prominent role in breast cancer-specific mortality but it does not fully explain the racial/ethnic disparities and continued research in genetic, societal, and lifestyle factors is warranted.

## Competing interests

The authors declare that they have no competing interests.

## Pre-publication history

The pre-publication history for this paper can be accessed here:

http://www.biomedcentral.com/1471-2407/13/449/prepub
